# Cytokine and Oxylipin Production in Resting and LPS‐Stimulated Monocytes From Americans of African Ancestry Are Influenced by *ALOX5* Promoter Tandem Repeat Polymorphisms

**DOI:** 10.1155/mi/8899139

**Published:** 2026-05-15

**Authors:** Ryan G. Snodgrass, Charles B. Stephensen, Patrice Armstrong, Hooman Allayee, Isabel F. Snodgrass, Kevin D. Laugero, John W. Newman

**Affiliations:** ^1^ Diet, Microbiome, and Immunity Research Unit, United States Department of Agriculture-Agricultural Research Services-Western Human Nutrition Research Center, Davis, California, USA, usda.gov; ^2^ Department of Nutrition, University of California Davis, Davis, California, USA, ucdavis.edu; ^3^ Department of Medicine, David Geffen School of Medicine at UCLA, Los Angeles, California, USA, ucla.edu; ^4^ West Coast Metabolomics Center, Genome Center, University of California Davis, Davis, California, USA, ucdavis.edu; ^5^ Obesity and Metabolism Research Unit, United States Department of Agriculture-Agricultural Research Services-Western Human Nutrition Research Center, Davis, California, USA, usda.gov

**Keywords:** 5-LOX, ALOX5, COX, cyclooxygenase, cytokine, monocyte, oxylipin

## Abstract

The arachidonate 5‐lipoxygenase (*ALOX5*) specificity protein 1 (Sp1) promoter tandem repeat polymorphism is associated with enhanced cardiovascular disease (CVD) risk. However, a functional understanding of these variants in immune cells central to disease development remains limited. We investigated oxylipin and cytokine production in resting and lipopolysaccharide (LPS)‐stimulated CD14+ monocytes from individuals carrying promoter variants and common alleles of *ALOX5*. Compared to monocytes from subjects carrying two common five‐repeat alleles (55 genotype), resting monocytes from individuals carrying one or more *ALOX5* deletion (d) alleles (d5 and dd genotypes) produced increased levels of IL‐1β, IL‐6, TNF‐ɑ, and IL‐10 but lower quantities of putative trihydroxyeicosapenataenoic acid (TriHEPE) isomers detected with the same mass transition as resolvin E1 (RvE1) but slightly longer retention times. With the common 55 genotype, TriHEPEs increased as IL‐6, IL‐10, and TNF‐ɑ production increased. This positive relationship between TriHEPEs and cytokines was diminished in monocytes with truncated *ALOX5* alleles. In response to LPS, monocytes from individuals with d5 and dd genotypes produced higher levels of IL‐1β, IL‐6, and TNF‐ɑ but not IL‐10, as well as increased quantities of cyclooxygenase (COX) products 11‐hydroxyeicosatetraenoic acid (11‐HETE), prostaglandin E2 (PGE_2_), thromboxane B_2_ (TxB_2_), prostaglandin F2α (PGF_2α_), prostaglandin E1 (PGE_1_), and prostaglandin B2 (PGB_2_) compared to monocytes from individuals with the common 55 genotype. The observed changes in monocyte inflammatory mediator production provide a plausible link for the association of *ALOX5* deletion alleles and CVD risk.

**Trial Registration:** Clinical trial: registered on ClinicalTrials.gov (Identifier: NCT00536185)

## 1. Introduction

Several studies have reported that arachidonate 5‐lipoxygenase (*ALOX5*) specificity protein 1 (Sp1) promoter tandem repeat polymorphisms are associated with greater risk of cardiovascular disease (CVD), including atherosclerosis, coronary artery disease (CAD), and myocardial infarction [1–6]. The arachidonate 5‐lipoxygenase (5‐LOX) enzyme encoded by the *ALOX5* gene is central to the biosynthesis of proinflammatory lipid mediators that initiate and sustain inflammation [7]. Prominently expressed in immune cells of myeloid origin including monocytes, macrophages, granulocytes, mast cells, and dendritic cells, 5‐LOX catalyzes the conversion of the unesterified n‐6 (omega‐6) polyunsaturated fatty acid (PUFA) arachidonic acid (AA) into 5‐hydroperoxyeicosatrienoic acid (5‐HpETE), a precursor to multiple bioactive lipids including 5(S)‐HETE, 5‐oxo‐ETE, and class 4 leukotrienes (LTs) [7, 8]. These eicosanoids facilitate inflammatory and allergic responses through increasing vascular permeability, recruiting immune cells such as neutrophils to sites of inflammation or immune activity, and inducing bronchoconstriction [7, 9]. While AA is the preferred substrate, 5‐LOX also metabolizes oxidized AA derivatives as well as the n‐3 (omega‐3) PUFA eicosapentaenoic acid (EPA) and docosahexaenoic acid (DHA) into specialized proresolving mediators (SPMs) with putative roles in immune modulation, tissue repair, and the resolution of inflammation [8, 10].

Due to the pleiotropic effects of eicosanoids in physiology and immune regulation, inappropriate or imbalanced 5‐LOX activity can thus lead to loss of homeostasis and contribute to the pathogenesis of chronic inflammatory diseases [7, 11]. The role of LT B_4_ (LTB_4_) and cysteinyl LTs in the pathogenesis of chronic inflammatory diseases including asthma, rheumatoid arthritis, and atherosclerosis is well described [9, 11, 12]. Through activation of their respective cognate membrane–bound G protein–coupled receptors (GPRs), LTs promote neutrophil chemotaxis, adhesion of monocytes to vascular endothelial cells, promote vascular smooth muscle constriction, and increase vascular permeability in postcapillary venules [13, 14]. While 5‐LOX plays a central role in the biosynthesis of proinflammatory lipid mediators, it also plays a less understood role in resolving inflammation through the production of EPA‐derived E‐series (RvE) and DHA‐derived D‐series (RvDs) resolvins [10]. Of the many reported RvEs, resolvin E1 (RvE1) displays potent actions protecting against leukocyte‐mediated tissue injury and excessive proinflammatory responses [8, 15]. Similar to RvEs, RvDs including RvD1 and RvD2 also contribute to inflammation resolution by stimulating phagocytosis and efferocytosis, inhibiting IL‐1β secretion, and facilitating wound healing [10]. Reduced biosynthesis of RvEs and RvDs was reported in several inflammatory diseases, suggesting that imbalances in proinflammatory and proresolving mediators may contribute to chronic inflammatory disease [16–18].

The *ALOX5* core promoter region is GC‐rich and contains eight GC boxes, of which five are arranged in tandem [19]. These GC boxes are functional binding sites for the transcription factors Sp1 and early growth response‐1 (Egr‐1) and are essential for basal transcriptional activity. The most common allele observed in populations consists of five tandem GC boxes, with less frequent naturally occurring variants comprising either deletion of one or two Sp1‐binding sites or the addition of one Sp1‐binding site to the common five‐repeat allele [1, 20, 21]. The clinical relevance of these variants is well‐documented, where, for example, individuals carrying two variant *ALOX5* promoter alleles have increased carotid intima‐media thickness and are at elevated risk of CAD and myocardial infarction compared to subjects carrying the common five‐repeat allele. Deletion alleles were strongly associated with CAD in an Indian population and increased the risk of myocardial infarction in Danish men [3, 4]. While the clinical importance of *ALOX5* promoter tandem repeat polymorphisms has garnered attention for its potential role in chronic disease, the functional implications of these variants in immune cells central to disease development remain limited. Given that circulating monocytes are major producers of 5‐LOX–derived oxylipins and an essential cell type to the development of atherosclerosis, the aim of the present study was to investigate the functionality of peripheral blood monocytes from individuals with *ALOX5* promoter tandem repeat polymorphisms.

## 2. Participants and Methods

Samples for the experiments described in this study came from the baseline study visit of a randomized, double‐blind, placebo‐controlled intervention trial originally designed to examine the effect of n‐3 fatty acid supplementation on CVD risk factors in participants with different *ALOX5* gene variants [22]. The project was approved by the institutional review boards of the University of California, Davis, and Alta Bates Summit Medical Center. Written informed consent was obtained from all study participants.

### 2.1. Participants

Healthy adults (*n* = 112) who self‐identified as African American, Black, or of African ancestry were recruited into the study from three California cities (Davis, Sacramento, and Oakland), as previously reported [22, 23]. Potential subjects who reported a physician‐diagnosed chronic inflammation–related disease, including CVD, hypertension, diabetes, or a lipid disorder that required regular use of anti‐inflammatory or lipid‐lowering medication, were excluded. Subjects with an abnormal result on a standard chemistry panel, lipid panel, or complete blood count that suggested underlying disease were also excluded and referred to their physician for further evaluation. Other exclusions are listed in the Supporting Information of our previous publication [22].

### 2.2. Genotyping

Genotyping of the *ALOX5* promoter repeat polymorphism was performed from genomic DNA isolated from buccal swabs using previously described methods [1] and confirmed using lymphocyte genomic DNA.

### 2.3. Blood Draw

At the baseline visit (week 0), 80 mL of blood was drawn from the antecubital vein into sodium‐heparin tubes after an overnight fast of 12 h duration. Blood was mixed well and stored at room temperature until processing within 4 h at the Western Human Nutrition Research Center in Davis, California.

### 2.4. Plasma Preparation and Monocyte Isolation

Plasma preparation and monocyte isolation were performed as described previously [22]. Briefly, blood was centrifuged at 1500 g for 10 min at 25°C. Plasma was pooled from all tubes for each subject and centrifuged at 2500 g for 20 min at 20°C to remove the platelets. Plasma was stored at −80°C. The buffy coat was removed and used for monocyte isolation. Ficoll step gradients were used to separate the peripheral blood mononuclear cells (PBMCs) and granulocytes (using Histopaque 1119 and 1077; Sigma, St. Louis, MO). Monocytes were separated from the PBMCs by positive selection using CD14 MicroBeads with an LS magnetic column (Miltenyi Biotec, Auburn, CA). Purity of monocytes was assessed by flow cytometry analysis using PE‐labeled anti‐CD14 (Miltenyi) and isotype control antibody and by FACSCalibur flow cytometry (Becton Dickinson, San Jose, CA). Viability was assessed by Trypan blue exclusion and always exceeded 84%. The mean viability was 97.4 ± 2.6%.

### 2.5. Monocyte Cultures

Purified monocytes (1 × 10^6^ cells/mL) were cultured at 37°C in 5% CO2 using RPMI 1640 complete medium supplemented with 10% heat‐inactivated autologous plasma. Paired supernatants were collected 20 h after stimulation with 1 ng/mL lipopolysaccharide (LPS) (List Biological Laboratories, CA) and endotoxin‐free water.

### 2.6. Cytokine Analysis

IL‐1β, IL‐6, TNF‐ɑ, and IL‐10 in monocyte supernatants were simultaneously quantified using a human cytokine multiplex assay from Meso Scale Discovery (MSD; Rockville, MD) and the MSD Sector Imager 2400 (MSD) according to the manufacturer’s instructions.

### 2.7. Oxylipin Analysis

Nonesterified oxylipins in cell culture media were isolated by solid phase extraction and quantified by ultraperformance liquid chromatography‐tandem mass spectrometry against authentic standards using isotopically labeled internal standards as previously described [24, 25]. Briefly, cell culture supernatants (0.2 mL) were enriched with 5 µL of 0.2 mg/mL butylated hydroxytoluene/EDTA in 1:1 (v/v) methanol:water and 12 deuterated oxylipin surrogates including d4‐6‐keto‐PGF1α, d4‐prostaglandin F2α (PGF_2α_), d4‐PGD_2_, d4‐LTB_4_, d8‐12(S)‐HETE, and d4‐5‐HETE (Cayman Chemical, Ann Arbor, MI). A complete list of internal standards, their mass transitions, and retention times are included in Supporting Information 1: Table S1. Residues in supernatants were then trapped on 60 mg Oasis HLB polymeric matrix solid phase extraction cartridges (Waters Corp., Milford, MA), washed, and eluted with 0.5 mL of methanol followed by 2.0 mL of ethyl acetate into tubes containing 2 µL of glycerol. Samples were dried under vacuum and reconstituted with 100 µL of 400 nM cyclohexylurido‐dodecanoic acid (Cayman Chemical, Ann Arbor, MI) in methanol and filtered by centrifugation at 0.2 µm through polyvinylidene fluoride membranes. Analytes were separated by reversed‐phase chromatography with a 2.1 × 150 mm Acquity BEH C18 column (Waters Corp, Milford, MA) and quantified by negative mode electrospray ionization on a Sciex 4000 QTRAP (Sciex, Framingham MA) tandem mass spectrometer run in multireaction monitoring (MRM) mode (Waters Corp.). Oxylipins and deuterated surrogates were quantified using internal standard ratio response methodologies measured against a minimum 5‐point calibration curve bracketing all reported concentrations. Putative trihydroxyeicosapenataenoic acid (TriHEPE) metabolites detected in the RvE1 MRM collection window were quantified as RvE1. Samples (one in 20) were analyzed in replicate to assess the analytical precision.

### 2.8. Statistical Analysis

Variable clustering and statistical analyses were performed using JMP Pro 16.1.0 (Cary, NC, USA). Cytokine and oxylipin data were Johnson‐transformed prior to analysis due to non‐normal distribution. A mixed model was applied to determine effects on oxylipin production, functional oxylipin clusters, and cytokine production. The model included genotype as a between‐subject effect, treatment (untreated and LPS‐stimulated conditions) as a repeated within‐subject effect, and genotype x treatment interaction. A two‐way interaction suggested a treatment‐dependent effect of genotype (or genotype‐dependent effect of treatment). Upon identifying a significant genotype or treatment effect or genotype x treatment interaction, a post hoc pairwise comparison of least squares means (LSMeans) was performed using Student’s *t*‐test. In follow‐up to the mixed model for individual oxylipins including cluster 1 oxylipins, which identified significant differences across genotypes following LPS stimulation but not in the resting state, *ALOX5* variants were pooled for further analysis. Each component was examined using a standard least squares linear model. LSMeans and associated standard errors were calculated for both groups, with statistical significance determined via *F*‐test (*α* = 0.05). Because the genotype effect contained only two levels, no post hoc comparisons were necessary. In follow‐up to the mixed model for cytokines, which identified significant main effects of treatment and genotype, LSMeans and their associated standard errors were calculated for each genotype at rest and following LPS stimulation. Differences between treatment conditions and among the three genotypes were then determined using the Student’s *t*‐test. To determine whether the influence of genotype and treatment on oxylipin production varied by the extent of cytokine production in the basal (untreated condition) state, a follow‐up set of mixed models was applied. Independent variables included the categories of genotype (between‐subject) and treatment (within‐subject, untreated, and stimulated conditions) and the continuous variable of either basal IL‐1β, IL‐6, IL‐10, or TNF‐a. The model terms consisted of genotype, treatment, cytokine, genotype x treatment, genotype x cytokine, treatment x cytokine, and genotype x treatment x cytokine. To better understand the nature of a three‐way interaction, we estimated the expected value of oxylipin for each combination of genotype and treatment level (untreated and LPS‐stimulated conditions) at selected levels of the evaluated cytokine (mean and 90th and 10th percentiles across all subjects), using the LSMeans. Final assembly and preparation of all figures were done using GraphPad Prism10, v.10.2.1 and CorelDRAW Essentials 2021 (Corel Corporation, Ottawa, Canada).

## 3. Results

### 3.1. Subject Characteristics

The clinical characteristics of the 112 study subjects in this secondary analysis are shown in Table 1. Of the total, 30 subjects carried two common alleles defined as five Sp1 element tandem repeats in the *ALOX5* promoter and referred to as the “55” genotype. Fifty‐three subjects carried the “d5” genotype consisting of a deletion (d) variant and one common (“5”) allele, and 29 subjects carried two deletion variants referred to as the “dd” genotype. Sixty eight percent of participants were female, ages 20–59 years old, and BMI from 18.8 to 40.5 kg/m2. Subject age, sex, and BMI did not differ by genotype. Additional study subject characteristics and genotype distributions have been reported previously [22, 23].

**Table 1:** Demographic data of all participants and grouped by *ALOX5* genotypes (“55,” “d5,” and “dd”).

Variables	All	“55”	“d5”	“dd”	*p*‐Value
*n*	112	30	53	29	—
Sex, % female (*n*)	67.9 (76)	60.0 (18)	67.9 (36)	75.9 (22)	0.1799
Age (years)	35.5 ± 11.6	36.4 ± 12.9	36.6 ± 12.5	32.4 ± 7.8	0.2796
BMI (kg/m^2^)	27.7 ± 4.6	26.7 ± 3.5	28.3 ± 4.6	27.7 ± 5.6	0.1847

*Note:* Values are mean ± SD unless otherwise indicated. Age and BMI were transformed prior to comparison among genotypes by ANOVA. *p*‐Value for sex comparison among genotypes was computed by chi‐square.

### 3.2. Monocyte‐Derived Oxylipins

**Figure 1:**
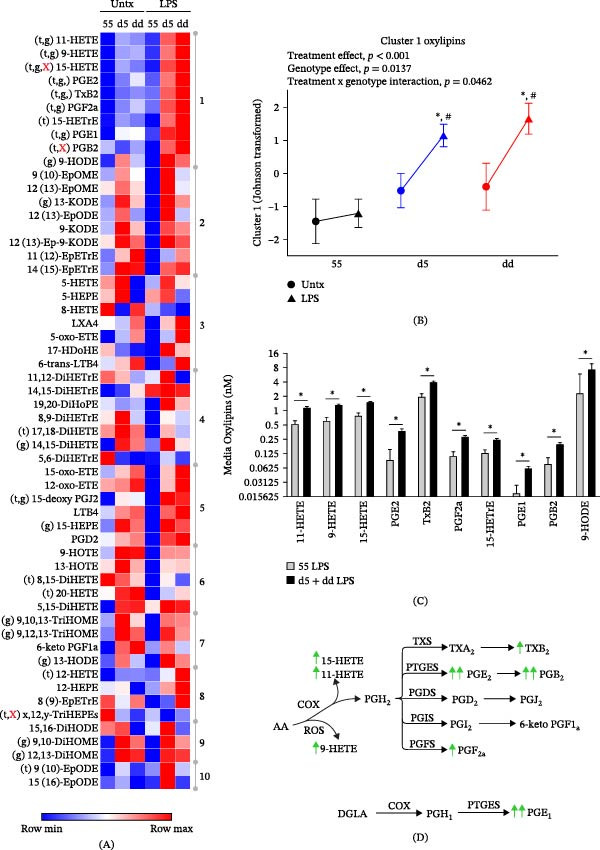
Oxylipin production in monocytes from subjects with *ALOX5* promoter tandem repeat polymorphisms. (A) Heatmap plot and cluster analysis of Johnson‐transformed mean oxylipins in supernatants of peripheral CD14+ monocytes isolated from subjects with *ALOX5* 55, d5, and dd genotypes left untreated or stimulated with 1 ng/mL LPS for 20 h. Repeated measures mixed model yielded significant main effects of genotype (55, d5, and dd; (g) *p*  < 0.05), treatment (untreated and LPS stimulation; (t) *p*  < 0.05), and genotype‐by‐treatment interactions ((X) *p*  < 0.05). (B) Follow‐up assessment of genotype‐by‐treatment interactions for cluster 1 oxylipins showing significant differences compared to within‐genotype untreated ( ^∗^ *p* < 0.05) and 55 LPS (#*p* < 0.05). (C) Mean ± SEM of cluster 1 oxylipins in supernatants of LPS‐stimulated 55 monocytes and d5+dd monocytes ( ^∗^ *p* < 0.05). (D) Increased conversion of AA and DGLA to COX‐derived oxylipins and autoxidative 9‐HETE in LPS‐stimulated d5 and dd monocytes compared to 55 monocytes. Abbreviations: AA, arachidonic acid; COX, cyclooxygenase; DGLA, dihomo‐γ‐linolenic acid; ROS, reactive oxygen species.

**Figure 2:**
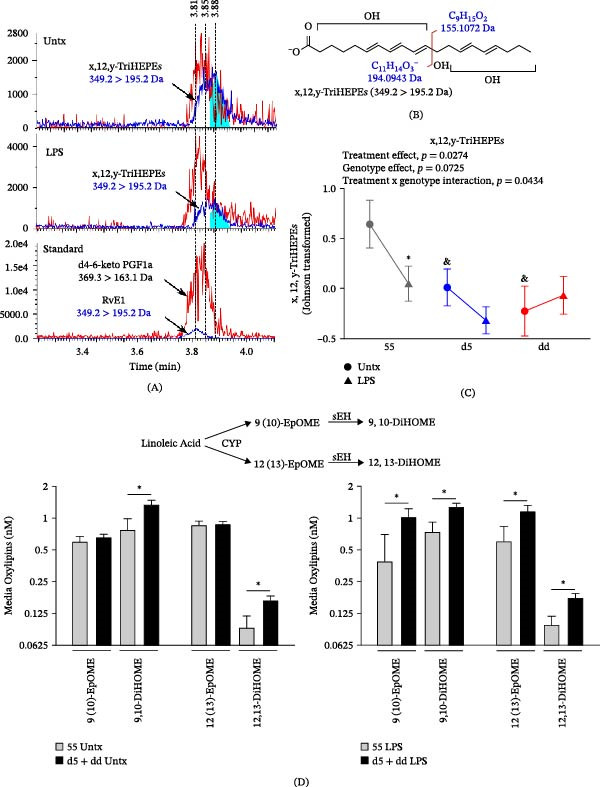
Putative x,12,y‐TriHEPEs and linoleic acid metabolite production in monocytes from subjects with *ALOX5* promoter tandem repeat polymorphisms. (A) Representative chromatograms of the putative TriHEPEs in supernatants of untreated (Untx) and LPS‐stimulated monocytes observed in the RvE1 acquisition window. Overlays in red of the closest eluting internal standard, deuterated 6‐ketoPG1Fa are provided to synchronize retention times. The width of the post‐RvE1 eluting signal and a subtle cavitation present in the untreated samples suggests the presence of at least two peaks, as indicated by the peak times and shading. (B) Shown is a conservative speculation of the structural nature of these unknowns. Based on the detection in the 349.2 > 195.2 Da mass transition, we consider 12‐hydroxylation of the EPA backbone likely. Further, the molecule must contain two additional oxygens, one of which occurs between positions 3 and 11 to allow the 195.2 fragment generation. Therefore, one oxygen must occur between carbon 13 and 20. (C) Follow‐up assessment of genotype‐by‐treatment interactions for x,12,y‐TriHEPEs showing significant differences compared to within‐genotype untreated ( ^∗^ *p* < 0.05) and 55 untreated (&*p* < 0.05). (D) Mean ± SEM of linoleic acid‐derived oxylipins in supernatants of untreated and LPS‐stimulated 55 monocytes and d5+dd monocytes ( ^∗^ *p* < 0.05). Abbreviations: CYP, cytochrome P450; RvE1, resolvin E1; sEH, soluble epoxide hydrolase.

To investigate potential functional differences among *ALOX5* promoter alleles, we first measured oxylipin production in monocytes isolated from the three genotype groups. Classical CD14+ monocytes isolated from the peripheral blood of study subjects were left untreated or stimulated with 1 ng/mL of LPS for 20 h. Cell culture supernatants were collected, and oxylipins were measured by mass spectrometry. Targeted analysis detected prostaglandins, mono‐, di‐, and trihydroxylated fatty acids derived from both n‐3 and n‐6 PUFAs, including products of cytochrome P450 (CYP), cyclooxygenase (COX), lipoxygenase (LOX), and nonenzymatic autoxidation. Variable clustering of all measured oxylipins in untreated and LPS‐stimulated monocyte supernatants produced 10 unique clusters (Figure 1A). Clusters and their oxylipin components were then characterized using repeated measures mixed models to assess the influence of the *ALOX5* genotype (55, d5, and dd) and treatment (untreated and LPS stimulation). A comprehensive list of treatment‐dependent genotype differences or genotype‐dependent treatment differences is shown in Supporting Information 2: Table S2. Cluster 1 explained the largest overall proportion of variance (14.1%) and was comprised of 10 oxylipins including autoxidation products (i.e., 9‐HETE), as well as COX‐derived (e.g., prostaglandin E2 [PGE_2_] and thromboxane B_2_ [TxB_2_]) and 15‐LOX–derived oxylipins (e.g., 15‐HETE). Metabolites in this cluster did not differ by genotype in the resting state but were elevated by LPS treatment. Notably, subjects with d5 and dd genotypes produced more oxylipins in response to LPS stimulation than those with the 55 genotype (P_treatment x genotype_ = 0.0462; Figure 1B–D). Cluster 2 explained 10.6% of the variance and was comprised of eight oxylipins including epoxides and ketones that were modestly elevated in the dd or d5 variants, with or without LPS stimulation. Cluster 3 oxylipins were exclusively LOX‐derived, including five of seven from 5‐LOX, and did not differ by genotype or treatment, as was generally the case for AA‐, EPA‐, and DHA‐derived dihydroxy fatty acids in cluster 4. Cluster 5 contained a mixture of compounds, including oxo‐fatty acids, LTB_4_, PGD_2_, and the PGD_2_ metabolite 15‐deoxy‐PGJ_2_, that showed borderline increases with the variant *ALOX5* genotypes. Clusters 6 and 7 contained compounds including the alpha‐linolenate–derived HOTEs and linoleate‐derived 13‐HODE and TriHOMEs that were elevated with dd and d5 genotypes (cluster 7; P_genotype_ = 0.0003) but unaffected by treatment. Cluster 8 comprised a suite of 12‐LOX metabolite and putative 12‐hydroxylated TriHEPEs. These TriHEPEs were serendipitously detected as they showed the same mass transition as the trihydroxylated EPA metabolite RvE1 and eluted within the signal collection window, with slightly longer retention times (Figure 2A). Based on the detection in the 349.2 > 195.2 Da mass transition, we consider a 12‐hydroxylation of the EPA backbone likely (Figure 2B). Further, the molecule must contain two additional oxygens, one occurring between positions 3 and 11 to allow the 195.2 Da fragment generation and a second likely occurring between carbon 13 and 20. Alternatively, if the 12‐hydroxyl was reduced to a ketone and one double bond was desaturated, as occurs in LTB_4_ metabolism, the resulting dihydro‐oxo‐DiHEPE would also produce the necessary precursor ion mass. However, it is unclear if the 195.2 Da isomer would be generated upon fragmentation. For the sake of this manuscript, we refer to these collective TriHEPE isomers as x,12,y‐TriHEPEs. Although a decidedly weak member of the cluster (*r* ^2^ within its own cluster = 0.21), x,12,y‐TriHEPE levels were elevated in supernatants of resting monocytes from subjects with the 55 genotype (0.98 nM ± 0.16 SEM) compared to d5 (0.69 nM ± 0.12 SEM) and dd (0.39 nM ± 0.17 SEM) genotypes, and as shown in Figure 2C, were suppressed by LPS stimulation (*P*_treatment x genotype_ = 0.0434; 55—0.56 nM ± 0.08 SEM; d5—0.45 nM ± 0.07; dd—0.48 nM ± 0.09). Clusters 9 and 10 contained linoleic acid (LA) and alpha‐linolenic acid (ALA) metabolites produced by the CYP and soluble epoxide hydrolase (sEH) pathway. Both cluster 9 diols, 9,10‐DiHOME and 12,13‐DiHOME, exhibited a significant main effect of genotype and were increased in supernatants of d5 and dd monocytes compared to 55 monocytes both at rest and following LPS stimulation (Figure 2D).

### 3.3. Monocyte‐Derived Cytokines

**Figure 3:**
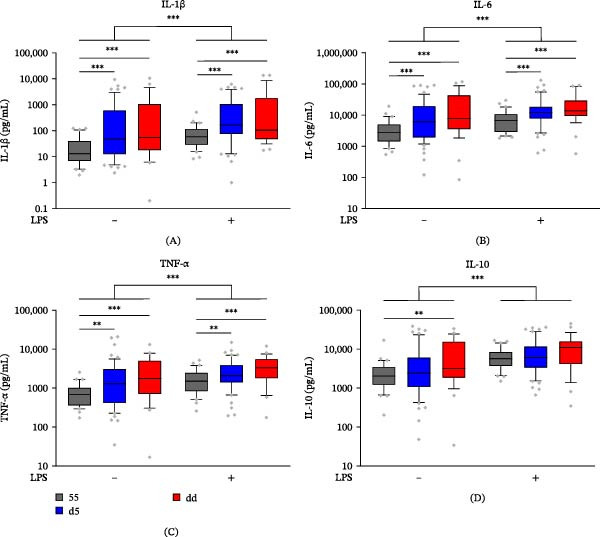
Cytokine production in monocytes from subjects with *ALOX5* promoter tandem repeat polymorphisms. (A) IL‐1β, (B) IL‐6, (C) TNF‐ɑ, and (D) IL‐10 in supernatants of CD14+ monocytes isolated from subjects with *ALOX5* 55, d5, and dd genotypes left untreated or stimulated with 1 ng/mL LPS for 20 h. Box and whisker plots show median, 10th, 25th, 75th, and 90th percentiles. Points above and below whiskers are shown as individual points. Significant treatment differences and differences compared to 55 genotypes are indicated by an ( ^∗^);  ^∗∗^ *p* < 0.01 and  ^∗∗∗^ *p* < 0.001.

To assess the influence of *ALOX5* genotype and treatment on cytokine production, we next measured levels of IL‐1β, IL‐6, TNF‐ɑ, and IL‐10 in the supernatants of untreated and LPS‐stimulated CD14+ monocytes. All cytokines exhibited a strong main effect of treatment (*p* < 0.0001) with increased levels following LPS stimulation (Figure 3A–D and Supporting Information 3: Table S3). We found lower concentrations of cytokines with the 55 compared to d5 and dd genotypes in untreated and LPS‐stimulated systems with the main effect of genotype for IL‐1β (*p* = 0.0005), IL‐6 (*p* = 0.0007), and TNF‐ɑ (*p* = 0.0084) but not IL‐10 (*p* = 0.11). While IL‐10 levels in subjects with the 55 genotype were lower than the dd group in the resting state (*p* = 0.0029), levels did not differ by genotype following LPS stimulation. These results show that, apart from IL‐10, both resting and LPS‐stimulated monocytes from subjects carrying deleted *ALOX5* Sp1 promoter tandem repeat variants produce larger amounts of cytokines compared to monocytes from subjects carrying two common five‐repeat alleles.

### 3.4. Cytokine and Oxylipin Interactions

**Figure 4:**
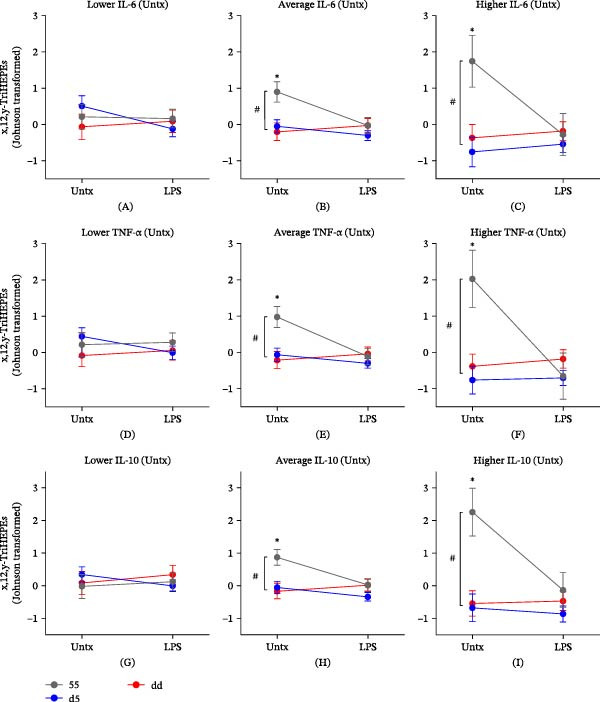
Influence of genotype and treatment on x,12,y‐TriHEPE production varies by the extent of cytokine production in the resting state. Estimation of the expected value of Johnson‐transformed x,12,y‐TriHEPE (±SEM) concentrations for each combination of *ALOX5* genotype (55, d5, and dd) and treatment level (untreated and LPS stimulation) at selected levels (mean and 90th and 10th percentiles across all subjects) of IL‐6 (A, B, C), TNF‐ɑ (D, E, F), and IL‐10 (G, H, I) using least square means. A follow‐up assessment of the three‐way interaction for x,12,y‐TriHEPE using Student’s *t*‐test showed differences between untreated and LPS‐stimulated 55 monocytes at mean and 90th percentiles ( ^∗^ *p* < 0.05) but not at the 10th percentiles and differences between untreated 55 monocytes and untreated d5 and dd monocytes at mean and 90th percentiles (#*p* < 0.05) but not at the 10th percentiles.

Since multiple studies have shown that cytokines and oxylipins can coregulate each other [26–28], we next explored the extent to which the observed differences in basal cytokine production in monocytes from subjects carrying *ALOX5* Sp1 promoter tandem repeat variants impact oxylipin production. Building on our repeated measures mixed model in which we identified significant genotype‐by‐treatment interactions for 15‐HETE, prostaglandin B2 (PGB_2_), and x,12,y‐TriHEPEs (Figure 1A), we then added an additional fixed effect of basal cytokine to assess its impact on 15‐HETE, PGB_2_, and x,12,y‐TriHEPE production. Our results show that the x,12,y‐TriHEPEs, but not 15‐HETE or PGB_2_, appeared to be influenced by a significant three‐way interaction between *ALOX5* genotype, treatment, and basal cytokine levels for IL‐6 (*p* = 0.0178), TNF‐ɑ (*p* = 0.0044), and IL‐10 (*p* = 0.0276) but not IL‐1β (*p* = 0.2186). This indicates that the influence of genotype and treatment on x,12,y‐TriHEPE production varies by the extent of cytokine production in the resting state. To better understand the nature of this three‐way interaction, we estimated the expected value of x,12,y‐TriHEPEs for each combination of genotype and treatment (untreated and LPS stimulation) at selected levels of IL‐6, TNF‐ɑ, and IL‐10 (mean and 90th and 10th percentiles across all subjects), using the LSMeans. The x,12,y‐TriHEPE concentrations for each combination of genotype and treatment level at the 10th, mean, and 90th percentile of basal IL‐6, TNF‐ɑ, and IL‐10 are displayed in Figure 4. In resting monocytes that produce lower levels of IL‐6, TNF‐ɑ, or IL‐10, x,12,y‐TriHEPE production is similar between all genotypes and does not change in response to LPS stimulation (Figure 4A,D,G). However, for resting monocytes, which produce average levels of IL‐6, TNF‐ɑ, or IL‐10, monocytes from subjects with the 55 genotype produce significantly more x,12,y‐TriHEPEs compared to dd and d5 monocytes (Figure 4B,E,H). Upon LPS stimulation, these higher basal x,12,y‐TriHEPE concentrations in 55 monocytes decrease to levels comparable to those observed in dd and d5 cells. This pattern becomes even more pronounced in the group of monocytes producing the highest levels of basal IL‐6, TNF‐ɑ, and IL‐10 (Figure 4C,F,I). Taken together, these results suggest a unique and complex interaction between basal cytokine production and x,12,y‐TriHEPE biosynthesis in peripheral blood monocytes that may be impaired in subjects carrying deleted *ALOX5* Sp1 promoter tandem repeat variants.

## 4. Discussion

While numerous studies have reported that *ALOX5* promoter tandem repeat polymorphisms are associated with a greater risk of CVD [1–6], the functional implications of these variants in immune cells central to disease development remain limited. Given that circulating monocytes are essential cell types to the development and exacerbation of atherosclerosis, we investigated the functionality of peripheral blood monocytes from individuals with *ALOX5* promoter tandem repeat polymorphisms. In the basal state, we found that monocytes with truncated *ALOX5* alleles simultaneously produced increased amounts of proinflammatory cytokines and lower amounts of TriHEPE isomers compared to monocytes from subjects carrying the two common alleles with 5 Sp1 repeats. We also found that in response to LPS stimulation, monocytes from subjects carrying *ALOX5* deletion alleles sustained their heightened proinflammatory cytokine secretion and produced increased levels of oxylipins derived from COX metabolism compared to 55 monocytes. Collectively, these findings indicate that monocytes harboring *ALOX5* variant alleles exhibit increased inflammatory activity, offering a biological explanation for their known association with CVD.

Oxylipins generated enzymatically by LOXs, COXs, and CYPs can exert various pro‐ and anti‐inflammatory effects [8]. While eicosanoids such as prostaglandins and LTs can facilitate proinflammatory and allergic responses, other oxylipins can play significant roles in immune modulation, tissue repair, and resolving inflammation [10, 29]. Adding to the complexity, in its canonical activity as an oxylipin synthase, 5‐LOX participates in complex biosynthesis pathways, forming precursors of lipoxins, resolvins, and LTs, which can exhibit potent anti‐inflammatory and proresolving properties [8, 30]. Moreover, accumulating evidence suggests that a decreased production of proresolving mediators contributes to inflammatory disease progression [17, 18, 31, 32]. At rest, we observed that monocytes from subjects with deleted *ALOX5* promoter tandem repeats produced lower amounts of x,12,y‐TriHEPEs and increased levels of 9,10‐ and 12,13‐DiHOME. Although supraphysiological levels of 12,13‐DiHOME have been shown to suppress LPS‐induced IL‐6 and TNF‐ɑ production [33], the implications of increased 9,10‐ and 12,13‐DiHOME production in monocytes are not clear. While not observed here, RvE1 and RvD1‐4, trihydroxylated EPA and DHA oxylipins, exhibit potent anti‐inflammatory and atheroprotective properties in the pico‐nanogram range [34]. In humans, vulnerable atherosclerotic plaque regions were shown to have significantly less 5‐LOX–derived SPMs compared with stable plaque regions [17], while hypertensive patients and those with CAD reportedly have lower levels of SPMs, including RvE1 [31, 35]. Notably, resolvins can attenuate NF‐κB signaling and proinflammatory cytokine production through binding cell surface GPRs, including ERV1/ChemR23, LTB_4_ receptor 1 (BLT1), GPR32, N‐formyl peptide receptor 2 (FPR2), and GPR18 [10, 32, 36]. In addition, many RvE1 metabolites have an RvE1‐like capacity to resolve inflammation [35, 37]. While its activity was not investigated, human monocytes can also produce the 349.2 Da 10,11‐dihydro‐12‐oxo‐RvE1 [35]. Consistent with prior reports showing that cytokines and oxylipins can coregulate each other [26, 38, 39], we also observed that the influence of *ALOX5* genotype on x,12,y‐TriHEPE production varied by the extent of IL‐6, TNF‐ɑ, and IL‐10 production in the resting state. In unstimulated monocytes with the 55 genotype, x,12,y‐TriHEPEs increased as IL‐6, TNF‐ɑ, and IL‐10 production increased, a response strongly diminished in monocytes from subjects with *ALOX5* deletion alleles. Interestingly, the resolvin receptor ERV1/ChemR23 is also strongly upregulated by IL‐6 in human monocytes [40]. If the observed x,12,y‐TriHEPEs can interact with ERV1/ChemR23, the loss of a dependent regulatory feedback on IL‐6 may partially explain why proinflammatory cytokine production in monocytes from subjects with deleted *ALOX5* promoter tandem repeats remains elevated. Confirmation of such behavior will take substantial effort in the future.

In response to LPS, monocytes from subjects with *ALOX5* deletion alleles also had enhanced production of proinflammatory cytokines IL‐1β, IL‐6, and TNF‐ɑ as well as oxylipins associated with COX metabolism but not 5‐LOX metabolites. Our results showing enhanced cytokine production align with a recent report by Blokhina et al. showing that classical monocytes derived from individuals with premature CAD produced more IL‐1β, IL‐6, and TNF‐ɑ compared to age‐matched subjects without CAD [41]. Additionally, Pande et al. reported that monocytes from individuals with peripheral artery disease have higher expression levels of IL‐6 than healthy subjects [42]. With respect to the increased oxylipin levels, 11‐hydroxyeicosatetraenoic acid (11‐HETE), PGE_2_, TxB_2_, PGF2a, prostaglandin E1 (PGE_1_), and PGB_2_ formation are all COX‐dependent, while 15‐HETE, 15‐HETrE, and 9‐HODE can have either autoxidative‐, COX‐, or 15‐LOX‐dependent production [8, 43–45]. Levels of the AA metabolite 9‐HETE were also elevated in *ALOX5* variant monocytes. While 9‐HETE is considered a nonenzymatic peroxidation product and marker of oxidative stress [46, 47] as such, it simply reports on the presence of reactive oxygen species (ROS) and not its source. In fact, COX‐dependent ROS production has been established [48] and is consistent with our current interpretation of the results. While elevated F2‐isoprostane was not detected, this is consistent with COX‐dependent ROS production in monocytes [49]. Human COX is present in two isoforms. COX‐1 is constitutively expressed in many cells and is thought to be responsible for the formation of lipid mediators involved in homeostatic functions, while COX‐2 is rapidly and transiently induced in response to inflammatory stimuli such as cytokines and endotoxins [8, 50]. Following the synthesis of COX‐derived endoperoxide PGG_2_ and PGH_2_ from free AA, the metastable intermediate PGH_2_ is rapidly converted into biologically active prostaglandins or thromboxanes by various cell‐specific enzymes [8]. In addition to detecting increased levels of TxB_2_ and PGF2a in supernatants of LPS‐stimulated monocytes with *ALOX5* deletion alleles, PGE_2_ and its nonenzymatic dehydration product PGB_2_ were also strongly upregulated compared to monocytes from subjects carrying two five‐repeat alleles. LPS exposure activates phospholipase A_2_, elevating intracellular free fatty acid levels [51]. Therefore, the increase in COX metabolite levels relative to the basal state observed across genotypes following LPS stimulation is likely driven by increased substrate availability rather than changes in COX expression.

Our observation of increased COX‐derived metabolite production in LPS‐stimulated *ALOX5* variant monocytes is particularly significant, as 5‐LOX was recently found to regulate COX‐2 expression in monocytic cells independent of its catalytic activity [52, 53]. While noncanonical 5‐LOX functions are well‐documented [30, 54], Kreiss et al. demonstrated that 5‐LOX inversely regulates *PTGS2* (COX‐2) transcription by directly associating with active chromatin regions within the COX‐2 promoter [52]. It is mechanistically intriguing that *ALOX5* variant monocytes—despite possessing total 5‐LOX protein levels similar to common allele monocytes—exhibit increased inflammatory cytokines and COX metabolites alongside lower levels of AA‐ and EPA‐derived 5‐LOX metabolites (5‐HETE and 5‐HEPE) upon Ca2+ ionophore stimulation [22]. Consequently, the discrepancy in inflammatory mediator production between genotype groups—despite equivalent total protein levels—warrants further investigation into canonical and noncanonical 5‐LOX functions as well as its subcellular localization (cytosolic vs. nuclear) in *ALOX5* variant monocytes.

We acknowledge several limitations associated with this study. First, as these data were derived from the baseline visit of a randomized, double‐blind, placebo‐controlled intervention trial, we were unable to acquire additional biological material for follow‐up experiments. This particularly restricted our ability to characterize the structure of the putative TriHEPEs. While these metabolites are consistent with a closely eluting RvE1 isomer and exhibit fragmentation patterns typical of other polyhydroxy oxylipins [55], the absence of spectra for similar monocyte‐derived EPA metabolites (e.g., 10,11,‐dihydro‐12‐oxo‐RvE1) or commercially available standards necessitates further investigation to confirm the structure and biological activity. Second, although myeloid cells including monocytes and macrophages express the enzymatic machinery (5‐LOX, 15‐LOX, and COX) required to synthesize the detected oxylipins, we cannot rule out that these metabolites or their precursors originated from the autologous plasma used in the monocyte cultures. Third, while negative selection isolation techniques are often preferred for functional assays, we utilized positive selection with anti‐CD14 antibodies; this approach may have influenced monocyte responsiveness to LPS, as previously reported [56]. Also, while we focused our analyses on the three‐repeat and four‐repeat variants, it is still possible that variants in linkage disequilibrium with these promoter alleles could also influence the expression of *ALOX5* and/or other genes at the locus. Finally, while COX‐dependent ROS production is consistent with the observed elevation in 9‐HETE levels, other sources of intracellular ROS production including CYP activity or mitochondrial leakage cannot be ruled out.

In summary, our results indicate that monocytes derived from subjects with truncated *ALOX5* promoter tandem repeats produce increased levels of proinflammatory cytokines and exhibit an altered oxylipin profile compared to monocytes from subjects carrying two common five‐repeat alleles. Given that inflammation is a critical driver of all stages of atherosclerosis, the observed changes in both stimulated and unstimulated monocyte inflammatory mediator production provide a plausible biological explanation for the association of *ALOX5* variant alleles with CVD risk.

## Author Contributions

Charles B. Stephensen, John W. Newman, Patrice Armstrong, and Hooman Allayee designed the research. Charles B. Stephensen, John W. Newman, Patrice Armstrong, and Hooman Allayee conducted the research. Ryan G. Snodgrass, John W. Newman, Isabel F. Snodgrass, and Kevin D. Laugero analyzed the data. Ryan G. Snodgrass, John W. Newman, Isabel F. Snodgrass, and Kevin D. Laugero interpreted results of experiments. Ryan G. Snodgrass and John W. Newman prepared figures. Ryan G. Snodgrass wrote the manuscript. Ryan G. Snodgrass, John W. Newman, and Kevin D. Laugero edited and revised the manuscript.

## Funding

This work was supported by NIH (Grants AT003411 and HL079353) and by US Department of Agriculture (USDA) ‐ Agricultural Research Service (ARS) (Grants 2032‐10700‐002‐00D and 2032‐10700‐003‐00D).

## Disclosure

All authors read and approved the final manuscript.

## Conflicts of Interest

The authors declare no conflicts of interest.

## Supporting Information

Additional supporting information can be found online in the Supporting Information section.

## Supporting information


**Supporting Information 1** Table S1: Oxylipin internal standards with retention times and mass transitions.

**Supporting Information 2** Table S2: Least squares means and standard error of Johnson‐transformed oxylipins from untreated and LPS‐stimulated monocytes stratified by cluster and ALOX5 promoter tandem repeat polymorphism.

**Supporting Information 3** Table S3: Least squares means and standard error of Johnson‐transformed cytokines from untreated and LPS‐stimulated monocytes stratified by ALOX5 promoter tandem repeat polymorphism.

## Data Availability

The data that support the findings of this study are available from the corresponding author upon reasonable request.
